# Determinants of Diet Quality in Young Football Players from Poznań, Poland

**DOI:** 10.3390/nu17172760

**Published:** 2025-08-26

**Authors:** Ewa Bryl, Anna Demuth, Joanna Ratajczak, Urszula Czerniak, Justyna Płoszka, Magdalena Lewandowska, Agnieszka Bilska, Katarzyna Antosiak-Cyrak

**Affiliations:** 1 Department of Human Biological Development, Faculty of Sport Sciences, Poznan University of Physical Education, 61-871 Poznań, Poland; demuth@awf.poznan.pl (A.D.); jratajczak@awf.poznan.pl (J.R.); czerniak@awf.poznan.pl (U.C.); 2 Student Scientific Club at the Department of Swimming and Water Lifesaving, Faculty of Sport Sciences, Poznan University of Physical Education, 61-871 Poznań, Poland; 3 Calculation Centre, Poznan University of Physical Education, 61-871 Poznań, Poland; lewandowska@awf.poznan.pl; 4 Department of Food and Nutrition, Faculty of Health Sciences, Poznan University of Physical Education, 61-871 Poznań, Poland; bilska@awf.poznan.pl; 5 Department of Swimming and Water Lifesaving, Faculty of Sport Sciences, Poznan University of Physical Education, 61-871 Poznań, Poland; antosiak-cyrak@awf.poznan.pl

**Keywords:** youth athletes, football players, diet quality, nutrition knowledge, dietary habits, nutritional education, family influence

## Abstract

Background/Objectives: Proper nutrition is crucial for the growth, development, and performance of young football players. Despite higher nutritional needs, physically active adolescents often have a suboptimal diet. This study assessed the diet quality of youth football players aged 11–16 from Poznań, Poland, focusing on the frequency of consuming health-promoting and non-healthy food groups. Methods: Participants were Football Championship School students. A total of 78 boys were analyzed and divided into early (11–13 years) and middle adolescence (14–16 years) groups. Dietary behaviours, nutritional knowledge, physical activity, screen time, and family affluence were assessed using a validated questionnaire (SF-FFQ4PolishChildren). The pro-Healthy Diet Index (pHDI) and non-Healthy Diet Index (nHDI) were calculated based on the frequency of food consumption. Results: The key predictors of the pro-Healthy Diet Index (pHDI) were the Cole index (β = −0.39; *p* < 0.001), subjective self-assessment of dietary habits (β = 0.23; *p* = 0.023), and the level of nutritional knowledge (β = 0.22; *p* = 0.030), explaining 25% of the variance in pHDI. In early adolescence, the Cole index was the main predictor (β = −0.51, *p* < 0.001, R^2^ = 32%), whereas in middle adolescence, self-assessment of dietary habits (β = 0.49, *p* = 0.002) and nutritional knowledge (β = 0.34, *p* = 0.03, R^2^ = 30) were the strongest predictors. Despite high levels of physical activity and positive self-assessment, only 1.28% of participants met all key criteria for a healthy diet. Screen time was negatively correlated with physical activity and positively associated with energy drink consumption. Conclusions: The results highlight a discrepancy between declared knowledge and actual behaviours, emphasizing the need for targeted, multi-level interventions involving families and coaches to improve dietary practices in young athletes.

## 1. Introduction

Proper nutrition constitutes a fundamental factor determining the physical development and exercise capacity of young athletes, including football players [1]. An adequately balanced diet plays a pivotal role in optimising metabolic, regenerative, and adaptive processes in the body in response to intensive physical exertion [2]. Nevertheless, the overall quality of the diet among young athletes remains an issue that warrants in-depth examination, particularly in relation to the consumption patterns of specific food groups and the various factors influencing dietary choices within this population.

In Poland, the National Institute of Public Health-National Institute of Hygiene (NIPH-NIH) is responsible for developing official dietary guidelines for children and adolescents. These recommendations are presented in a clear and accessible format for both children and their caregivers through the Healthy Nutrition and Lifestyle Pyramid [3]. The guidelines emphasize the importance of consuming an appropriate number of meals per day, eating breakfast, staying hydrated (primarily through water consumption), and incorporating vegetables, fruits, whole grain products, dairy, lean meats, eggs, legumes, and plant-based fats into the daily diet. At the same time, they advocate limiting the intake of animal fats, sugary beverages, sweets, salty snacks, and fast food. As its name indicates, the Healthy Nutrition and Lifestyle Pyramid addresses not only dietary choices but also essential lifestyle behaviours conducive to maintaining good health. It promotes daily physical activity, adequate sleep, reduced screen time, and avoidance of excessive salt consumption. These recommendations form the foundation for the development of healthy eating habits, and their effective implementation should be reinforced through comprehensive nutrition education.

Despite the availability of such national guidelines, proper nutrition continues to be a fundamental factor requiring further attention, particularly in the context of young athletes. This discrepancy between recommended guidelines and actual dietary behaviours highlights the need for a deeper understanding of their nutritional behaviours [4], particularly in relation to frequency of consumption of specific food groups and the various factors that shape their eating habits.

One of the key determinants of diet quality is the level of nutritional education [5], which in Poland is still insufficient, especially among children and adolescents. Limited awareness of the principles of healthy eating can adversely affect dietary habits, which is of particular importance in the case of young athletes, for whom an adequate intake of nutrients is essential for proper growth and athletic performance [6]. Although the introduction of nutritional education as a compulsory component of the primary school curriculum is planned, there remains a need for a more comprehensive analysis of its impact on the development of health-promoting eating behaviours.

The primary aim of this study is to evaluate the diet quality of young football players from Poznań, with particular emphasis on the frequency of consumption of various food groups. Furthermore, the analysis seeks to identify factors determining diet quality, with special consideration given to the role of nutritional education and sources of information regarding proper nutrition. The findings obtained may provide valuable insights into the development of effective educational strategies aimed at improving dietary habits among young athletes.

## 2. Materials and Methods

A cross-sectional observational study was conducted from February to April 2023. All participants were students off a Football Championship School with a specialization in football. In addition to the standard school curriculum, they took part in specialized football training, with a total weekly training time of 360–400 min (4–5 session per week). The recruited athletes competed at the highest league in their age categories, ranking among the top teams in Greater Poland region. Players represented three competitive groups (U11-U16), with each league comprising 16 teams (a total of 960 players; data from the Greater Poland Football Association). All students aged 11–16 attending the school were invited to participate. According to the World Health Organization classification, participants were divided into two categories: early adolescence (11–13 years) and middle adolescence (14–16 years) [7]. A total 81 players initially agreed to participate, of whom 78 boys aged 11–16 years were included the final analysis. Three participants were excluded due to missing data for key dietary variables, resulting in a response rate of 96.3%. All were football players training at local sports club in Poznań, a city in western Poland with a population of approximately 540,000 residents [8]. Recruitment to the team was based on physical fitness tests, observation of gameplay, and an assessment of psychological predispositions conducted by the coaching staff. All coaches held pedagogical qualifications (graduated from the University of Physical Education) and UEFA A or B coaching licenses. The questionnaire was completed independently by the participants at home and were checked for completeness by the researcher upon submission. Written informed consent was obtained from parents or legal guardians, and verbal assent was given by the participants. No financial or material incentives were provided for participation in the study.

Data on dietary behaviours, nutrition knowledge, lifestyle, sociodemographic variables, family affluence, and anthropometric parameters were collected using the standardised, self-administered questionnaire Short-Form Food Frequency Questionnaire for Polish Children (SF-FFQ4PolishChildren) [9], with a reference period of the previous year. The questionnaire consisted of 44 questions covering, among others, eating habits (including breakfast frequency and consumption of selected food groups), test questions assessing nutrition knowledge, information on physical activity, screen time, place of residence and the family’s material status (components of the Family Affluence Scale, FAS). The results were interpreted according to the guidance of the questionnaire authors.

Family affluence was assessed based on questions regarding car ownership, number of holiday trips, whether the child had their own room, and the number of computers, laptops, and tablets in the household. The obtained scores (range: 0–7) were summed and participants were categorised as having low (0–4 points), moderate (5–6 points), or high (7 points) family affluence.

Nutrition knowledge was evaluated based on the number of points (0–18) scored in the test included in the questionnaire. Participants were then assigned to one of three categories: low knowledge (0–4 points), moderate knowledge (5–7 points), or high knowledge (8–18 points).

Diet quality was assessed using the pro-Healthy Diet quality index (pHDI) and the non-Healthy Diet quality index (nHDI) [9], calculated based on the frequency of consumption of specific food groups (dairy products, fish, vegetables, and fruit for pHDI; fast food, sugar-sweetened carbonated beverages, energy drinks, and sweets for nHDI). The frequency of consumption was converted to a daily value and summed for each index, with a maximum possible score of 8. The results were expressed as a percentage score (0–100%). Scores were then categorised as low (<33.33%), moderate (33.33–66.66%), or high (≥66.66%) diet quality. Additionally, the regularity of breakfast consumption was analysed and categorised as regular (7 days/week) or irregular (4–6 days/week). Detailed instructions for calculating pHDI and nHDI are provided in the Supplementary File S1.

Lifestyle was assessed based on screen time, physical activity at school, physical activity during leisure time. Screen time was calculated based on the self-reported average number of hours per day and categorised: <2 h/day, 2–4 h/day, or ≥4 h/day. School and leisure time physical activity were assessed separately in three categories (low, moderate, vigorous).

Self-assessment of diet was assessed based on a question with four response options (definitely yes, rather yes, rather no, definitely no). For the purpose of analysis, the responses were grouped into two categories: correct (definitely yes and rather yes) and incorrect (definitely no and rather no).

Source of knowledge about health nutrition in sports were assessed through a multiple-choice question. Participants could select one or more options from the following list: I do not seek such knowledge, Internet, Friends, Coach, Parents, Dietician, Physician, and Books.

Body weight was measured using a RADWAG C315.60/150.OW-1 (Radom, Poland) accurate to 0.01 kg, under standard conditions: participants wore light sports clothing and no shoes. Body height was measured with an GMP Swiss Made anthropometer accurate to 0.1 cm, with the head positioned in the Frankfurt plane.

Body mass index (BMI) was calculated using the formula: BMI = body weight [kg]/(height [m]) ^2^. The Cole Index was then computed as follows: Cole Index = (BMI/BMI at the 50th percentile) × 100%. The results were categorized as follows: <90%—undernutrition; 91–110%—normal nutritional status; 111–120%—overweight; and >120%—obesity [10].

The frequency of consumption of foods considered health-promoting, such as dairy products, fish, vegetables, and fruit, was assessed according to the Healthy Eating Pyramid guidelines for children and adolescents [3]. In addition, the intake of foods with a negative impact on health, which should be limited, was analysed—namely fast food, sugar-sweetened carbonated beverages, energy drinks, and sweets. According to current recommendations, dairy products should be consumed several times per day, fish at least twice per week, and vegetables and fruit several times per day. The intake of highly processed foods and sugary drinks should be kept as low as possible.

All statistical analyses were performed using STATISTICA 13 (TIBCO Software Inc. 2017, Tulsa, OK, USA). The normality of the distribution of continuous variables was verified using the Shapiro–Wilk test. A significance level of *p* < 0.05 was adopted for all analyses. For variables with a normal distribution, Pearson’s correlation coefficient was calculated, for those not normally distributed and for ordinal variables, Spearman’s rank correlation was used. Correlation analyses were also performed separately in the age groups of early adolescence (11–13 years) and middle adolescence (14–16 years). To identify the determinants of diet quality, multiple linear regression models were constructed with the pro-Healthy Diet Index (pHDI) as the dependent variable. In the whole sample, only variables significantly correlated with the diet quality index (excluding those used to calculate the index) were included in the regression model. For subgroup analyses, the same set of variables identified as significant predictors in whole-group model was used.

## 3. Results

### 3.1. Demographic and Anthropometric Characteristics of Participants

The study included 78 young football players aged 11 to 16 years, with a equal number of participants in each age group: 39 individuals aged 11–13 years (early adolescence) and 39 individuals aged 14–16 (middle adolescence) [7]. The majority of respondents lived in urban areas (58.97%), while 41.03% came from rural areas. The Family Affluence Scale (FAS) indicated that 42.31% of the participants had a high family affluence level, 48.72% a moderate level, and only 8.97% a low level. The Cole index analysis indicated that 70.50% of players had normal weight, 20.51% were underweight, and 8.97% were overweight (Table 1).

### 3.2. Lifestyle Profile: Training Experience, Physical Activity and Screen Time

The majority of the players had been training football for at least 6 years (69.23%). The overall level of physical activity was high in half of the participants (50.00%) and moderate in 44.87%. Most participants reported a high level of physical activity both at school (60.25%) and during leisure time (80.77%).

Regarding screen time, for the majority of participants it ranged from 2 to 4 h per day (48.72%), while 33.33% reported spending less than 2 h per day, and 17.95% more than 4 h daily (Table 2).

### 3.3. Nutritional Knowledge, Dietary Habits, and Sources of Nutrition Information in Young Football Players

The participants’ nutrition knowledge was generally high—the mean score was 9.15 points (SD = 3.11), with as many as 71.79% classified as having high knowledge and only 7.69% as having low knowledge. The mean pro-Healthy Diet Index (pHDI) score was 2.68 (SD = 1.07), and none of the participants achieved a high level. A low diet quality was observed in 48.72% of participants, and a moderate quality in 51.28%. The non-Healthy Diet Index (nHDI) was low in all participants (mean = 0.58, SD = 0.49).

The main sources of knowledge about healthy eating were parents (75.64%), the Internet (50.00%), and coaches (48.72%); dietitians (16.67%) and physicians (10.26%) were indicated much less frequently. A total of 89.74% of participants rated their own diet as appropriate (Table 3).

### 3.4. Assessment of Compliance with Nutritional Recommendations

An analysis of the frequency of consumption of selected food groups showed that only a portion of the respondents met the basic guidelines of the Healthy Eating and Physical Activity Pyramid for children and adolescents. The most consistently followed recommendation was eating breakfast daily, reported by 85.90% of participants. However, for other food categories, compliance with the recommendations was significantly lower. Only 32.05% of participants declared consuming vegetables several times a day, meaning that nearly 68% did not meet this basic dietary guideline. Fruit consumption several times a day was reported by 46.15% of participants, indicating that more than half of the respondents (53.85%) did not follow the recommendation in this regard. Only 15.38% of participants ate fish at least twice a week. Dairy products were consumed several times a day by 23.08% of the respondents, while 76.92% consumed them less frequently than recommended. Limiting sweets consumption to once a week or less was reported by 46.15% of participants. Fast food was consumed less than once a week by 83.33% of the respondents, and sugary drinks by 62.82% in these two categories, the majority complied with the recommendations to limit highly processed foods. As many as 85.90% of participants avoided energy drinks entirely or consumed them very rarely.

In summary, only a small proportion of respondents met all the key principles of the Healthy Eating Pyramid (Table 4).

After counting the children who met all four dietary recommendations at the level recommended by the National Institute of Food and Nutrition, only 1 child (1.28%) in the analyzed sample achieved a satisfactory result. Eight children (10.26%) met any three recommendations. In contrast, the group that met only one or none of the Institute’s recommendations regarding health-promoting food consumption accounted for 37.18% (*n* = 29) and 29.49% (*n* = 23) of the sample, respectively (Table 5).

Unfortunately, the opposite situation occurs when it comes to solving the problem of processed products. Only two children (2.56%) from the analyzed sample excluded all 4 components that make up unhealthy diets (fast food, sweet desserts, sweets, energy drinks), and as many as 28.21% of children did not restrict consumption of any of the 4 product groups included in the recommendations (Table 5).

### 3.5. Correlations Between Diet Quality, Nutritional Knowledge, Lifestyle and Nutritional Variables

Statistically significant associations were observed between pHDI and nutritional knowledge (r = 0.25) and diet self-assessment (r = 0.29). This indicates that a higher level of nutritional knowledge and a positive diet self-assessment are associated with better diet quality. Additionally, statistically significant negative correlations were found between diet quality index and the Cole index (r = −0.30), suggesting that higher body mass (relative to age and sex) is associated with lower diet quality. The Cole index was negatively associated with the intake of vegetables (r = −0.23) and diary products (r = −0.25) and positively correlated with the consumption of energy drinks (r = 0.29). Nutritional knowledge was negatively correlate with energy drink consumption (r = −0.22). (Table 6).

Consumption of sweets was positively associated with fast food (r = 0.31), sweet beverages (r = 0.36) and interestingly vegetables intake (r = 0.24). Additionally, the intake of sweets was negatively correlated with fish consumption (r = −0.30). Energy drink consumption was correlated with sweet beverages intake (r = 0.32) and screen time (r = 0.27). Physical activity in leisure time was negatively correlated with screen time (r = −0.26) (Table 6).

In the group of children aged 11–13 years, classified as early adolescence, a statistically significant negative correlation was observed between the pro-Healthy Diet Index (pHDI) and the Cole index (r = –0.42). Positive correlations were found between the consumption of sweets and the consumption of fast food (r = 0.43), sweetened soft drinks (r = 0.47), as well as vegetables (r = 0.33) and fruit (r = 0.34). Surprisingly, fruit consumption also showed a positive correlation with screen time (r = 0.33) (Table 7).

In the group of children aged 14–16 years, representing middle adolescence, a statistically significant positive correlation was observed between the pro-Healthy Diet Index (pHDI) and self-assessed diet quality (r = 0.46). Additionally, self-assessed diet quality was positively correlated with the consumption of dairy products (r = 0.46) and with physical activity during leisure time (r = 0.37). Consumption of sweetened soft drinks was positively correlated with the intake of fast foods (r = 0.37), energy drinks (r = 0.36), and screen time (r = 0.36). Fast food consumption was also positively correlated with energy drink consumption (r = 0.39). Consumption of sweets was negatively correlated with fish intake (r = –0.35). Vegetable and fruit consumption were positively correlated (r = 0.51) (Table 8).

### 3.6. Determinants of Diet Quality Among Young Athletes in the Whole Group and Age Subgroups

In order to identify factors significantly predicting diet quality among children and adolescents in whole group, a forward stepwise regression analysis was conducted (Table 9). Only variables that were significantly correlated with the diet quality index were included in the regression model, excluding those used to calculate the index. The dependent variable was the pro-healthy diet index (pHDI), while the predictors included the Cole index, subjective assessment of dietary habits, and nutrition knowledge. The final model identified all three as statistically significant predictors: the Cole index (β = −0.39; *p* < 0.001), self-assessed diet quality (β = 0.23; *p* = 0.023), and nutrition knowledge (β = 0.22; *p* = 0.030). Altogether, the model was statistically significant and explained 25% of the variance in pHDI.

In the early adolescence group (11–13 years), the multiple linear regression model including the Cole index and nutritional knowledge explained 32% of the variance in pHDI. The Cole index was a significant negative predictor of pHDI (β = −0.51, *p* < 0.001), whereas nutritional knowledge showed a positive but not-significant association (β = 0.27, *p* = 0.06) (Table 9).

In the middle adolescence group (14–16 years), the multiple linear regression model including self-assessment of diet, nutritional knowledge, and the Cole index explained 30% of the variance in the pHDI. Self-assessment of diet was a significant positive predictor of pHDI (β = 0.49, *p* = 0.002), as was nutritional knowledge (β = 0.34, *p* = 0.03). The Cole index did not show a significant association with pHDI in this age group (β = −0.15, *p* = 0.31) (Table 9).

## 4. Discussion

Before interpreting the study findings, it is important to consider the context of school nutrition education in Poland. School-age youth in Poznań do not have guaranteed free meals (breakfast or lunches) at school, but they have the option to purchase meals in school cafeterias or bring food from home, as well as buy snacks from school kiosks. According to Polish regulations, the sale of “junk food” such as sweets, sugary carbonated drinks, chips, and similar products on school premises is prohibited (regulated by the Act on Food Safety and Nutrition by the Minister of Health Dz.U. 2016 poz. 1154) [11]. Although nutrition education is encouraged, a comprehensive and unified program dedicated solely to nutrition education in Polish school is still lacking. Instead, nutrition topics are integrated within other subjects such as physical education, biology, nature studies, educational hours, and safety education. These contextual factors provide an important background when considering dietary habits and nutritional knowledge in the study population.

The analysis revealed that significant predictors of the pro-healthy diet index (pHDI) among the children practicing football were: the Cole Index, subjective self-assessment of eating habits, and the level of nutritional knowledge. In the age stratified analyses, in the early adolescence group (11–13 years) the Cole index emerged as the sole significant predictor of pHDI, making it the main contributor to the explained variance in this subgroup. In contrast, in the middle adolescence group (14–16 years), diet quality was significantly associated with self-assessment of diet and nutritional knowledge. This pattern suggests that while younger children’s diet quality is more closely linked to anthropometric status, in older adolescents it appears to depend more on self-perception, dietary awareness, and nutritional knowledge.

In both the overall sample and particularly in early adolescence, a significant negative relationship was found between the Cole index and diet quality. This indicates that higher BMI-for-age is associated with less favorable dietary habits, where lower BMI is linked to better pHDI score. Although most participants had a normal body weight, the observed correlations suggest that lower levels of pro-healthy dietary behaviours may contribute to increases in body mass index, even at this early stage. This is a relevant public health concern, as meta-analyses indicate that obese children and adolescents are about five times more likely to remain obese in adulthood compared to their peers with normal body weight [12]. In other words, excessive body weight in childhood may constitute a potential risk factor for developing overweight or obesity in the future, and it is also linked to an increased risk of metabolic syndrome in adulthood [13]. Such findings highlight the importance of shaping healthy eating habits from early age and reinforce the need to prioritise nutritional education as a preventive strategy.

The study results demonstrate that a higher level of nutritional knowledge and a positive self-assessment of one’s own dietary habits are associated with better diet quality among young football players in Poznań, especially in the middle adolescence group. It can be assumed that children who are more knowledgeable about nutrition are more aware of the consequences of their daily dietary choices, which in turn contributes to more positive evaluations of their eating behaviours [14]. Such self-assessments may reinforce engagement in maintaining healthy habits and serve as a motivator for making further conscious dietary decisions [15].

However, nutritional knowledge alone appears to be insufficient to induce lasting changes in children’s dietary behaviours. A literature review published in 2021 reports a weak association between knowledge about nutrition and actual dietary practices, suggesting that knowledge does not automatically translate into healthier choices [16]. This is consistent with our observations—despite over 70% of the children demonstrating a high level of nutritional knowledge, none of them achieved a high score on the pro-healthy diet index. This finding emphasizes the need for a multifaceted approach to nutrition education that goes beyond mere knowledge transfer to also include practical skills development and environmental support.

An analysis of the frequency of consumption of specific food groups included in the pHDI further confirmed significant discrepancies between reported knowledge and actual dietary habits. While breakfast, an essential component of a healthy lifestyle, was regularly consumed by 85.9% of participants, adherence to other Healthy Eating Pyramid guidelines, such as adequate intake of vegetables, fruits, dairy products, and fish, was considerably lower. Only one child in the entire group declared consumption of all the recommended food groups with the advised frequency. This reflects that a high level of knowledge does not guarantee the automatic implementation of healthy dietary behaviours.

Attention should also be paid to the sources of nutritional knowledge used by children. In the study group, parents were most frequently indicated as the primary source of information, confirming their key role in shaping the dietary attitudes of young athletes [17]. As parents are responsible for food purchases and meal preparation, educational efforts should be directed not only toward children but also—if not primarily—toward their caregivers [18]. Supporting parents’ nutritional competence can have a real impact on their children’s diet quality [19]. A concerning finding is that 50% of the children identified the internet as a source of nutritional knowledge. Although there are credible and informative resources online such as those published by the National Center for Nutritional Education [20]—children may also access unverified sources, including social media, blogs, or video platforms. A lack of critical evaluation skills can lead to the reinforcement of incorrect nutritional beliefs and encourage poor dietary decisions [21].

Our observations align with broader research findings. For instance, Austrian researchers have also reported low dietary quality among children and adolescents, regardless of gender, age, or parental education level [5]. Similarly, studies on young Spanish footballers found that 54.8% did not adequately follow the principles of the Mediterranean diet [22]. Turkish data indicated that over 90% of surveyed athletes had a low-quality diet [23], while in Brazil, 72.7% of participants were classified as having poor dietary quality [24].

In contrast, results from Portugal are more encouraging, where 31.1% and 68.2% of young footballers followed the Mediterranean diet principles to a high and moderate degree, respectively [25]. These figures suggest the presence of factors that promote better dietary practices within this population. One possible explanation is effective and consistent nutrition education delivered through engaging and attractive formats. A good example is the “Football and Nutrition for Health” program, which after just 12 weeks contributed to increased nutritional knowledge and improved intake of fruits and fish among children training in football [26].

These findings point to the need for developing integrated educational programs that combine theory with practice [27]. Nutritional education should not be limited to providing information about healthy and unhealthy foods. Equally important are practical competencies such as meal planning, grocery shopping, preparing simple and nutritious meals, and understanding the role of nutrition in physical performance and recovery. This is particularly relevant for physically active children, whose nutritional needs are increased due to training-related demands.

Furthermore, educational efforts should be implemented through multiple channels—in the home, at school, in sports clubs, and with the involvement of professionals such as dietitians, coaches, and teachers [28]. Only such a comprehensive approach can effectively bridge the gap between knowledge and practice and lead to lasting improvements in the dietary behaviours of young athletes.

### Limitations

This study has several limitations that should be acknowledged. First, the use of a questionnaire-based method may be subject to reporting bias, including social desirability and recall errors. Second, dietary quality was assessed without quantitative evaluation of actual nutrient intake, which limits the ability to draw conclusions about specific dietary deficiencies or excesses. Third, the relatively small sample size reduces the statistical power of the findings and limits the generalizability of the results. Additionally, data on parental and family eating habits were not collected, although these factors are known to significantly influence children’s dietary behaviours and may act as potential confounders.

## 5. Conclusions

The key predictors of pro-healthy diet index among young football players from Poznań were the Cole index, subjective self-assessment of eating habits and nutritional knowledge. A higher Cole Index was associated with lower diet quality, whereas better self-assessed eating habits and higher level of nutritional knowledge were associated with improved diet quality. In age-stratified analyses, diet quality in the early adolescence group (11–13 years) was significantly associated with the Cole Index, while in the middle adolescence group (14–16 years) it was determined by self-assessment of diet and nutritional knowledge.

Despite a high level of physical activity, positive assessment of eating habits, and high nutritional knowledge, most young football players did not adhere to dietary recommendations. Only 1.28 participants met all key criteria for a healthy diet. A clear discrepancy was observed between the subjective assessment of nutritional knowledge and actual dietary behaviour.

Lifestyle factors, including physical activity and screen time, had a significant impact on diet quality and eating habits. A leisure- time physical activity was negatively correlated with screen time, while energy drink consumption was associated with longer screen exposure.

These findings provide valuable starting points for future intervention studies aimed at testing the effectiveness of targeted educational or behavioral programs designed to improve dietary habits among young athletes.

## Figures and Tables

**Table 1:** Characteristics of the study group—demographic and anthropometric.

Variable	*n*	%
Sample size	78	100
Age		
11–13 years old	39	50.00
14–16 years old	39	50.00
Residence		
Rural	32	41.03
Urban	46	58.97
FAS (level)		
Low	7	8.97
Moderate	38	48.72
high	33	42.31
Cole index	Mean = 97.92, SD = 10.25	
thin	16	20.51
normal weight	55	70.5
overweight	7	8.97

**Table 2:** Characteristics of the study group—physical activity, training experience and screen time among study participants.

Variable	*n*	%
Football training internship		
3–4 years	12	15.38
5–6 years	12	15.38
6 and more years	54	69.23
Physical activity level		
Low	4	5.13
Moderate	35	44.87
High	39	50.00
Physical activity at school		
Low	6	7.69
Moderate	25	32.05
High	47	60.25
Physical activity at leisure time		
Low	4	5.13
Moderate	11	14.10
High	63	80.77
Screen time		
<2 h	26	33.33
2–4 h	38	48.72
>4 h	14	17.95

**Table 3:** Characteristics of the study group—nutritional knowledge, dietary habits.

Variable	*n*	%
Nutrition knowledge	Mean = 9.15, SD = 3.11	
Low	6	7.69
Moderate	16	20.51
High	56	71.79
Pro-healthy diet quality index (pHDI)	Mean = 2.68, SD = 1.07	
Low	38	48.72
Moderate	40	51.28
High	0	0.00
Non-healthy diet quality index (nHDI)	Mean = 0.58, SD = 0.49	
Low	78	100.00
Medium	0	0.00
Self-assessment of diet		
Incorrect	8	10.26
Correct	70	89.74
Source of knowledge about healthy nutrition in sports		
Not seeking such knowledge	6	7.70
Internet	39	50.00
Friends	7	8.97
Coach	38	48.72
Parents	59	75.64
Dietician	13	16.67
Physician	8	10.26
Books	1	1.28

**Table 4:** Dietary habits and frequency of consumption of selected food groups.

Variable	*n*	%
Dietary habits		
Breakfast		
Every day (7/week)	67	85.90
Irregular (4–6/week)	11	14.10
Consumption of food groups		
Dairy products		
Several times a day	18	23.08
Less often	60	76.92
Fish		
Twice or more than week	12	15.38
Less often	66	84.62
Vegetables		
Several times a day	25	32.05
Less often	53	67.95
Fruit		
Several times a day	36	46.15
Less often	42	53.85
Fast food		
Less than once a week	65	83.33
More often	13	16.67
Sweet beverages		
Less than once a week	50	62.82
More often	29	37.18
Energy drinks		
Never or almost never	67	85.90
More often	11	14.10
Sweets		
Once a week or less	36	46.15
More often	42	53.85

**Table 5:** Number of fulfilled dietary recommendations pro-healthy and non-healthy behaviours.

	Pro-Healthy Behaviours		Non-Healthy Behaviours	
Number of Dietary Recommendations Met	*n*	%	*n*	%
0	23	29.49	2	2.56
1	29	37.18	5	6.41
2	17	21.79	23	29.49
3	8	10.26	26	33.33
4	1	1.28	22	28.21

**Table 6:** Correlations between Diet Quality, Nutritional Indicators, Lifestyle factors and Cole index in Whole Group.

	(1)	(2)	(3)	(4)	(5)	(6)	(7)	(8)	(9)	(10)	(11)	(12)	(13)	(14)
(1) Cole index														
(2) pHDI	−0.30 ^a^*													
(3) Nutritional knowledge	−0.08 ^a^	0.25 ^b^*												
(4) Self-assessment of diet	−0.07 ^a^	0.29 ^a^*	0.00 ^a^											
(5) Fast food consumption	−0.01 ^a^	0.03 ^a^	−0.03 ^a^	−0.13 ^a^										
(6) Sweet beverages consumption	−0.04 ^a^	−0.06 ^a^	−0.02 ^a^	0.02 ^a^	0.28 ^a^*									
(7) Energy drinks consumption	0.29 ^a^*	−0.13 ^a^	−0.22 ^a^*	−0.02 ^a^	0.19 ^a^	0.32 ^a^*								
(8) Sweets consumption	−0.12 ^a^	0.16 ^a^	0.10 ^a^	0.06 ^a^	0.31 ^a^*	0.36 ^a^*	−0.20 ^a^							
(9) Diary products	−0.25 ^a^*	0.64 ^a^*	0.23 ^a^*	0.10 ^a^	0.10 ^a^	−0.01 ^a^	−0.25 ^a^*	0.18 ^a^						
(10) Fish	−0.12 ^a^	0.32 ^a^*	−0.16 ^a^	0.02 ^a^	−0.04 ^a^	−0.13 ^a^	0.10 ^a^	−0.30 ^a^*	0.18 ^a^					
(11) Vegetables	−0.23 ^a^*	0.69 ^a^*	0.23 ^a^*	0.26 ^a^*	0.06 ^a^	0.00 ^a^	−0.13 ^a^	0.24 ^a^*	0.23 ^a^*	0.09 ^a^				
(12) Fruit	−0.02 ^a^	0.54 ^a^*	0.16 ^a^	0.27 ^a^*	−0.06 ^a^	−0.02 ^a^	0.08 ^a^	0.16 ^a^	0.10 ^a^	0.03 ^a^	0.36 ^a^*			
(13) FAS	0.07 ^a^	−0.07 ^a^	−0.11 ^a^	0.08 ^a^	−0.07 ^a^	−0.05 ^a^	0.02 ^a^	0.01 ^a^	−0.02 ^a^	0.02 ^a^	−0.01 ^a^	−0.08 ^a^		
(14) Screen time	0.07 ^a^	0.05 ^a^	−0.02 ^a^	−0.11 ^a^	0.03 ^a^	0.17 ^a^	0.27 ^a^*	0.13 ^a^	0.01 ^a^	−0.09 ^a^	−0.04 ^a^	0.16 ^a^	0.14 ^a^	
(15) PA at leisure time	0.07 ^a^	0.15 ^a^	−0.01 ^a^	0.20 ^a^	0.02 ^a^	−0.01 ^a^	−0.08 ^a^	0.03 ^a^	0.09 ^a^	0.11 ^a^	0.10 ^a^	−0.07 ^a^	0.03 ^a^	−0.26 ^a^*

TIBCO Software Inc. diet quality index, FAS—Family Affluence Scale, PA—physical activity, ^a^—Spearman rank correlation coefficient, ^b^—Pearson correlation coefficient, *—indicates statistically significant differences (*p* < 0.05).

**Table 9:** Linear regression model summary: pHDI as dependent variable.

**Model 1 All Group**				
**Variable**	**R^2^**	β*	**F**	***p*** **Value**
Model	0.25		8.41	<0.001
Cole index		−0.39		<0.001
Nutritional knowledge		0.23		0.023
Self-assessment of diet		0.22		0.030
**Early adolescence**				
**Variable**	**R^2^**	β*	**F**	***p*** **Value**
Model	0.32		8.65	<0.001
Cole index		−0.50		0.001
Nutritional knowledge		0.27		0.06
**Middle adolescence**				
Model	0.30		5.07	<0.05
Self-assessment of diet		0.49		0.002
Nutritional knowledge		0.34		0.03
Cole index		−0.15		0.31

**Table 7:** Correlations between Diet Quality, Nutritional Indicators, Lifestyle factors and Cole index in Early Adolescence.

	(1)	(2)	(3)	(4)	(5)	(6)	(7)	(8)	(9)	(10)	(11)	(12)	(13)
(1) Cole index													
(2) pHDI	−0.42 ^a^*												
(3) Nutritional knowledge	−0.02 ^a^	0.26 ^a^											
(4) Self-assessment of diet	0.13 ^a^	0.13 ^a^	0.29 ^a^										
(5) Fast food consumption	−0.16 ^a^	0.02 ^a^	−0.08 ^a^	−0.23 ^a^									
(6) Sweet beverages consumption	−0.07 ^a^	−0.002 ^a^	0.05 ^a^	−0.04 ^a^	0.29 ^a^								
(7) Sweets consumption	−0.11 ^a^	−0.19 ^a^	−0.05 ^a^	−0.01 ^a^	0.43 ^a^*	0.47 ^a^*							
(8) Diary products	−0.27 ^a^	0.64 ^a^*	0.26 ^a^	−0.20 ^a^	0.02 ^a^	0.08 ^a^	0.13 ^a^						
(9) Fish	−0.25 ^a^	0.40 ^a^*	−0.13 ^a^	−0.13 ^a^	−0.04 ^a^	−0.10 ^a^	−0.23 ^a^	0.25 ^a^					
(10) Vegetables	−0.26 ^a^	0.67 ^a^*	0.21 ^a^	0.22 ^a^	0.21 ^a^	−0.17 ^a^	0.33 ^a^*	0.20 ^a^	0.09 ^a^				
(11) Fruit	−0.06 ^a^	0.39 ^a^*	0.24 ^a^	0.30 ^a^	−0.07 ^a^	−0.03 ^a^	0.34 ^a^*	0.11 ^a^	−0.11 ^a^	0.25 ^a^			
(12) FAS	0.04 ^a^	−0.06 ^a^	0.04 ^a^	−0.12 ^a^	0.11 ^a^	−0.18 ^a^	−0.14 ^a^	−0.10 ^a^	−0.12 ^a^	0.06 ^a^	−0.007 ^a^		
(13) Screen time	−0.04 ^a^	0.27 ^a^	−0.03 ^a^	−0.11 ^a^	0.16 ^a^	−0.06 ^a^	0.18 ^a^	0.26 ^a^	0.05 ^a^	0.08 ^a^	0.33 ^a^*	0.06 ^a^	
(14) PA at leisure time	0.13 ^a^	0.07 ^a^	0.14 ^a^	0.07 ^a^	0.09 ^a^	−0.07 ^a^	0.003 ^a^	−0.01 ^a^	0.004 ^a^	0.02 ^a^	−0.18 ^a^	−0.07 ^a^	−0.40 ^a^

pHDI—Pro-healthy diet quality index, FAS—Family Affluence Scale, PA—physical activity, ^a^—Spearman rank correlation coefficient, *—indicates statistically significant differences (*p* < 0.05).

**Table 8:** Correlations between Diet Quality, Nutritional Indicators, Lifestyle factors and Cole index in Middle Adolescence.

	(1)	(2)	(3)	(4)	(5)	(6)	(7)	(8)	(9)	(10)	(11)	(12)	(13)	(14)
(1) Cole index														
(2) pHDI	−0.09 ^a^													
(3) Nutritional knowledge	−0.06 ^a^	0.13 ^b^												
(4) Self-assessment of diet	−0.01 ^a^	0.46 ^a^*	−0.27 ^a^											
(5) Fast food consumption	0.18 ^a^	0.03 ^a^	−0.06 ^a^	−0.01 ^a^										
(6) Sweet beverages consumption	0.09 ^a^	−0.14 ^a^	−0.02 ^a^	0.05 ^a^	0.37 ^a^*									
(7) Energy drinks consumption	0.37 ^a^*	−0.17 ^a^	−0.25 ^a^	−0.06 ^a^	0.39 ^a^*	0.36 ^a^*								
(8) Sweets consumption	−0.16 ^a^	0.08 ^a^	0.20 ^a^	0.14 ^a^	0.16 ^a^	0.31 ^a^	−0.24 ^a^							
(9) Diary products	−0.16 ^a^	0.62 ^a^*	0.10 ^a^	0.46 ^a^*	0.12 ^a^	−0.04 ^a^	−0.27 ^a^	0.16 ^a^						
(10) Fish	−0.02 ^a^	0.29 ^a^	−0.14 ^a^	0.18 ^a^	−0.004 ^a^	−0.26 ^a^	0.10 ^a^	−0.35 ^a^*	0.16 ^a^					
(11) Vegetables	−0.15 ^a^	0.67 ^a^*	0.24 ^a^	0.27 ^a^	−0.11 ^a^	−0.17 ^a^	−0.18 ^a^	0.13 ^a^	0.25 ^a^	0.09 ^a^				
(12) Fruit	−0.23 ^a^	0.73 ^a^*	0.12 ^a^	0.24 ^a^	−0.01 ^a^	−0.08 ^a^	0.04 ^a^	0.003 ^a^	0.05 ^a^	0.17 ^a^	0.51 ^a^*			
(13) FAS	−0.29 ^a^	−0.06 ^a^	−0.23 ^a^	0.27 ^a^	−0.23 a	−0.02 ^a^	0.01 ^a^	0.20 ^a^	0.11 ^a^	0.14 ^a^	−0.07 ^a^	−0.19 ^a^		
(14) Screen time	0.07 ^a^	−0.09 ^a^	0.04 ^a^	−0.13 ^a^	−0.01 ^a^	0.36 ^a^*	0.28 ^a^	0.13 ^a^	−0.15 ^a^	−0.28 ^a^	−0.12 ^a^	−0.05 ^a^	0.16 ^a^	
(15) PA at leisure time	−0.16 ^a^	0.24 ^a^	−0.11 ^a^	0.37 ^a^*	0.01 ^a^	−0.03 ^a^	−0.25 ^a^	0.10 ^a^	0.32 ^a^*	0.22 ^a^	0.23 ^a^	0.05 ^a^	0.14 ^a^	−0.21 ^a^

pHDI—Pro-healthy diet quality index, FAS—Family Affluence Scale, PA—physical activity, ^a^—Spearman rank correlation coefficient, ^b^—Pearson correlation coefficient, *—indicates statistically significant differences (*p* < 0.05).

## Data Availability

The data presented in this study is available on request from the corresponding author.

## Supplementary Materials

The following are available online at https://www.mdpi.com/article/10.3390/nu17172760/s1, File S1: Calculation of the pro-Healthy Diet Index (pHDI) and non-Healthy Diet Index (nHDI) according to Kowalkowska et al. [9].

## Abbreviations

The following abbreviations are used in this manuscript:

pHDI	pro-Healthy Diet Index
nHDI	non-Healthy Diet Index
UEFA	Union of European Football Associations
SF-FFQ4PolishChildren	Short-Form Food Frequency Questionnaire for Polish Children
FAS	Family Affluence Scale
BMI	Body Mass Index
PA	Physical Activity
