# Supplementary material for: Determinants of Diet Quality in Young Football Players from Poznań, Poland
Source: Nutrients. 2025 Aug 26;17(17):2760. doi: 10.3390/nu17172760 (PMC12430096; doi:10.3390/nu17172760)
Supplement: Supplementary file 1 [file nutrients-17-02760-s001.zip › nutrients-3770483-supplementary.pdf]

Supplementary Materials

Calculation of the pro-Healthy Diet Index (pHDI) and non-Healthy Diet Index (nHDI) according to Kowalkowska et al.

(*Reproducibility of a Short-Form, Multicomponent Dietary Questionnaire to Assess Food Frequency Consumption, Nutrition Knowledge, and Lifestyle (SF-FFQ4PolishChildren) in Polish Children and Adolescents*).

Step 1.

Identify food items for each index

- pHDI: dairy products, fish, vegetables, fruit
- nHDI: fast foods, sweetened soft drinks, energy drinks and sweets or confectionery.

Step 2

Assign frequency categories

Responses for each item are coded into daily frequency values:

- never/almost never = 0
- less than once a week = 0.06
- once a week = 0.14
- 2–4 times/week = 0.43
- 5–6 times/week = 0.79
- every day =1
- a few times a day = 2

Step 3

Calculate the sum of the daily frequencies separately for each index (pHDI, nHDI).

Step 4

Convert to percentage score

pHDI=(sum of pHDI daily frequencies / maximum possible score) *100

nHDI=(sum of nHDI daily frequencies / maximum possible score) *100

where: maximum possible score is number of items x highest daily frequency → 4x2=8

Step 5

Classify diet quality

Low: 0–33.32%
Moderate: 33.33–66.65%
High: 66.66–100%

Kowalkowska, J.; Wadolowska, L.; Hamulka, J.; Wojtas, N.; Czlapka-Matyasik, M.; Kozirok, W.; Bronkowska, M.; Sadowska, J.; Naliwajko, S.; Dziaduch, I.; et al. Reproducibility of a Short-Form, Multicomponent Dietary Questionnaire to Assess Food Frequency Consumption, Nutrition Knowledge, and Lifestyle (SF-FFQ4PolishChildren) in Polish Children and Adolescents. *Nutrients* **2019**, *11*, 2929, doi:10.3390/nu11122929.
